# Cross-species fertilization: the hamster egg receptor, Juno, binds the human sperm ligand, Izumo1

**DOI:** 10.1098/rstb.2014.0101

**Published:** 2015-02-05

**Authors:** Enrica Bianchi, Gavin J. Wright

**Affiliations:** Cell Surface Signalling Laboratory, Wellcome Trust Sanger Institute, Hinxton, Cambridge, UK

**Keywords:** fertilization, Izumo1, Juno, oocyte, sperm, zona-free hamster egg penetration assay

## Abstract

Fertilization is the culminating event in sexual reproduction and requires the recognition and fusion of the haploid sperm and egg to form a new diploid organism. Specificity in these recognition events is one reason why sperm and eggs from different species are not normally compatible. One notable exception is the unusual ability of zona-free eggs from the Syrian golden hamster (*Mesocricetus auratus*) to recognize and fuse with human sperm, a phenomenon that has been exploited to assess sperm quality in assisted fertility treatments. Following our recent finding that the interaction between the sperm and egg recognition receptors Izumo1 and Juno is essential for fertilization, we now demonstrate concordance between the ability of Izumo1 and Juno from different species to interact, and the ability of their isolated gametes to cross-fertilize each other *in vitro*. In particular, we show that Juno from the golden hamster can directly interact with human Izumo1. These data suggest that the interaction between Izumo1 and Juno plays an important role in cross-species gamete recognition, and may inform the development of improved prognostic tests that do not require the use of animals to guide the most appropriate fertility treatment for infertile couples.

## 1. Introduction

**Figure 1.**
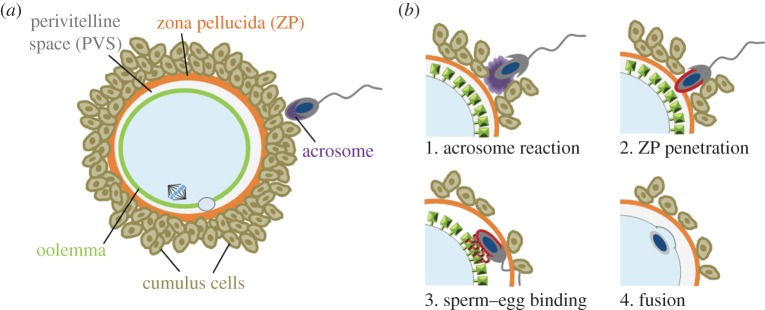
The cell biology of mammalian fertilization. (*a*) Diagram highlighting the main cellular features of both the mammalian sperm and egg important during fertilization. (*b*) Schematics showing the four main steps in mammalian fertilization. (1) Sperm undergo the acrosome reaction and pass through the egg investments: the cumulus cells and the zona pellucida. (2) The acrosome reaction triggers the re-localization of Izumo1 (red) from the acrosomal membrane to the sperm head plasma membrane. (3) Once within the perivitelline space, the now exposed Izumo1 ligand is able to interact with the egg receptor Juno (green) displayed on the oolemma. (4) Finally, the sperm and egg membranes fuse, fertilizing the egg.

In sexually reproducing species, the spermatozoon (sperm) and the egg are terminally differentiated haploid cells that are responsible for the propagation of genetic information from one generation to the next. When fertilization is successful, the sperm and the egg recognize and adhere to each other before fusing to form a new, genetically distinct diploid organism. The development of *in vitro* fertilization (IVF) assays has permitted a good cellular description of this process and has led to remarkable advances in fertility treatments enabling infertile couples to conceive. The first attempts to fertilize human eggs *in vitro* date back to the 1940s [1] and the first positive outcomes were obtained 30 years later [2]. An important advance in IVF research was the discovery that testicular sperm is unable to directly fertilize eggs, but must first be ‘capacitated’ within the female reproductive tract. Sperm capacitation culminates in the fusion of the acrosome—a vesicle present within the sperm head—with the plasma membrane so that fusion-competent sperm are often referred to as ‘acrosome-reacted’ (figure 1) [3]. Further progress in IVF research required finding a surrogate egg from a suitable animal model to substitute for human eggs which have understandably complex ethical issues connected with their use in research. One major barrier that prevents the fusion of isolated gametes from different species is the zona pellucida—a glycoprotein-rich coat that surrounds the ovulated oocyte—which exhibits species-specific interactions with sperm (figure 1) [4]. Interestingly, by removing the zona pellucida it was found that oocytes from the Syrian golden hamster (*Mesocricetus auratus*) had the unusual ability to fuse with acrosome-reacted sperm from several other mammalian species, including mouse, pig and human [5,6]. In 1976, Yanagimachi and colleagues [7] obtained swollen human sperm heads and male pronuclei in zona-free hamster oocytes and proposed using this procedure to evaluate human sperm function. The *z*ona-free *h*amster *e*gg *p*enetration assay—ZHEP—(also called *h*amster *z*ona-*f*ree *o*vum test, HZFO) initially raised great interest, but several studies have subsequently questioned its prognostic value [8,9]. Nevertheless, the hamster oocyte is able to fuse with sperm from different mammalian species, suggesting cross-species binding of the molecules that mediate sperm–egg recognition [6].

Izumo1 is a protein displayed on the surface of acrosome-reacted sperm that is necessary for the ability of the sperm to recognize and fuse with the egg (figure 1) [10]. Using an avid recombinant form of Izumo1 and an expression cloning approach, we recently identified the egg receptor for Izumo1 as a glycosylphosphatidylinositol (GPI)-anchored protein that was previously called folate receptor 4, but because it did not bind folate, we suggested renaming it Juno after the Roman goddess of fertility. Female *Juno*-deficient mice were infertile, implying that the Izumo1–Juno interaction is essential for fertilization [11]. Because Izumo1 and Juno are necessary for sperm–egg recognition, we reasoned that hamster Juno should bind to Izumo1 from other mammalian species. Here, we show using recombinant proteins that hamster Juno can directly interact with mouse, pig and human Izumo1, matching the ability of zona-free hamster eggs to fuse with human, mouse and pig sperm. These results provide a molecular explanation for the ability of gametes from different species to recognize and fuse with each other.

## 2. Material and methods

### (a) Juno and Izumo homologues

Protein sequences for Juno orthologues were mouse, *Mus musculus* (Uniprot accession number Q9EQF4); human, *Homo sapiens* (A6ND01) and golden hamster, *M. auratus* (NCBI Ref Seq XP_005084100.1). Izumo1 orthologue sequences were: *Homo sapiens* (Q8IYV9), *Mus musculus* (Q9D9J7) and *Sus scrofa* (F1RIQ7).

### (b) Recombinant protein production and purification

All proteins were expressed as soluble recombinant proteins where the entire predicted ectodomains were expressed from plasmid constructs made by gene synthesis (GeneArt), except mouse Juno where the ectodomain was amplified from a cDNA clone isolated as previously described [11]. The regions encoding the ectodomains of Juno and Izumo1 were flanked by unique NotI and AscI sites and subcloned into a derivative of the pTT3 expression vector [12] that contains a rat CD4 (Ig-like domains 3 and 4) tag for quantitation, and either an enzymatically biotinylatable peptide tag (‘bait’ vector), or a pentamerization domain from the rat cartilage oligomeric matrix protein (COMP) and β-lactamase (‘prey’ vector). Both bait and prey proteins also contained a C-terminal 6-His tag for purification [13]. Briefly, the proteins were expressed by transient transfection of HEK293E cells grown in suspension culture as previously described [14] and collected from the cell culture supernatant 6 days post-transfection. His-tagged proteins were purified from the culture supernatants by affinity chromatography on HisTrap HP columns (GE Healthcare) using an ÄKTAxpress (GE Healthcare) according to the manufacturer's instructions.

### (c) Extracellular protein interaction screening by AVEXIS

Bait and prey proteins were normalized to activities that have been previously shown to detect transient interactions [12] and screened using the ELISA-based AVEXIS methodology as described in [14]. Briefly, biotinylated bait proteins were immobilized on streptavidin-coated 96-well microtitre plates (Nunc) and washed with HBS. Normalized β-lactamase-tagged preys were incubated for 1 h, the wells were washed with HBS and finally 125 µg ml^−1^ of the β-lactamase substrate, nitrocefin, was added. Absorbance values were measured at 485 nm on a Pherastar Plus (BMG Laboratories). A bait protein consisting of the CD4d3+4 tag alone was used as the negative control. All steps were done at room temperature. The assays were repeated three times using independent protein preparations.

## 3. Results

### (a) Identification of hamster Juno

**Figure 2.**
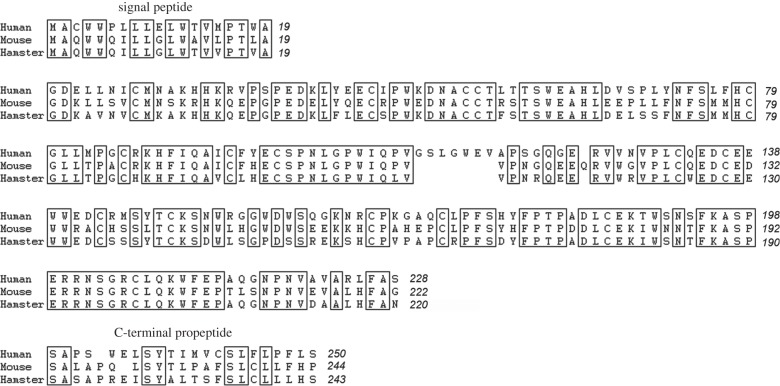
Alignment of the extracellular amino acid sequences of hamster Juno with the mouse and human orthologues. The amino acid sequence of the hamster (*M. auratus*) Juno was aligned with the human (*Homo sapiens*) and mouse (*Mus musculus*) orthologues and identical amino acids boxed. The signal peptide and the predicted C-terminal propeptide are indicated.

To determine whether human Izumo1 can bind hamster Juno, we decided to employ a protein interaction assay developed in our laboratory called AVEXIS (for AVidity based EXtracellular Interaction Screen), which detects direct binary interactions between recombinant soluble ectodomains expressed in mammalian cells [12]. The assay was purposefully designed to detect highly transient binding events which are a common feature of extracellular interactions mediated by cell surface receptor proteins [15]. The assay detects direct binding events between soluble recombinant proteins expressed as either monomeric biotinylated baits, which are captured on streptavidin-coated microtitre plates, and then systematically probed for interactions with pentamerized β-lactamase-tagged preys. Prey pentamerization is achieved through the use of a 46 amino acid sequence from the rat COMP which increases the overall binding avidity such that even very transient interactions can be detected by hydrolysis of a colorimetric β-lactamase substrate. We have previously shown that the AVEXIS assay can robustly detect interactions with half-lives less than 0.1 s with low false positive rates [12]. The affinity of the mouse Izumo1–Juno interaction was shown to be extremely weak with a half-life of approximately 0.5 s and can be detected by AVEXIS [11]. It would be reasonable to expect that the binding affinity of a cross-species interaction would be even weaker than this, necessitating the use of a sensitive assay. To identify the hamster Juno protein sequence so that a recombinant form could be expressed, we used the BLAST search tool [16] and the mouse Juno sequence to search a draft genome sequence of the Syrian golden hamster (*M. auratus*) that was recently deposited in publically accessible databases. A single candidate was identified that shared a high degree (more than 60%) of protein sequence identity with the mouse and human orthologues. Similar to the mouse and human sequences, hamster Juno had clearly identifiable expected protein features including a predicted N-terminal signal sequence peptide and a potential GPI modification site followed by a C-terminus propeptide which would both be cleaved from the mature protein (figure 2).

### (b) Hamster Juno binds human Izumo1

**Figure 3.**
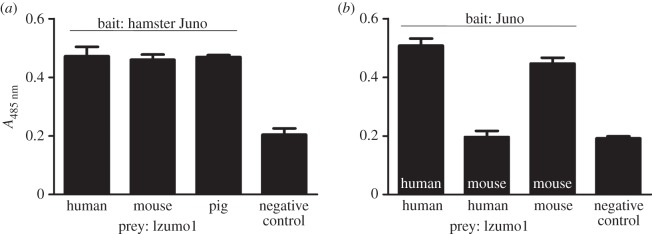
Human Izumo1 directly interacts with hamster, but not mouse Juno. (*a*) The hamster Juno biotinylated ‘bait’ protein was immobilized on streptavidin-coated microtitre plates and tested for its ability to bind human, mouse and pig Izumo1 by AVEXIS. Clear binding was observed with Izumo1 orthologues from all species relative to a negative control. (*b*) Human Izumo1 prey proteins were probed for interactions with either human or mouse Juno proteins presented as baits. Human Izumo1 interacted with human but not mouse Juno. The positive interaction between mouse Juno and mouse Izumo1 demonstrated that the mouse Juno bait protein was functional. Negative controls were the rat CD4 tag alone. Bar graphs represent mean ± s.d., *n* = 3.

We used the AVEXIS assay to test cross-species Izumo1–Juno binding, which requires expressing the entire ectodomains of both Izumo1 and Juno as soluble recombinant proteins in mammalian cells as an enzymatically monobiotinylated ‘bait’ or pentamerized, enzyme tagged ‘preys'. The hamster Juno protein was expressed as a bait and captured in individual wells of a streptavidin-coated microtitre plate before probing it for interactions with the Izumo1 orthologues from human, mouse and pig presented as preys. Using this approach, we observed that hamster Juno was able to interact directly with all three Izumo1 orthologues, including human (figure 3*a*). These binding reactions match the known fusing ability of acrosome-reacted sperm from these species with zona-free hamster oocytes [5,6]. Furthermore, it is known that acrosome-reacted human sperm is unable to fuse with zona-free mouse oocytes [17]. Given the essential requirement for the Izumo1–Juno interaction for fertilization we would therefore predict that mouse Juno should not interact with human Izumo1. To test this, human and mouse Juno were presented as immobilized baits and probed for interactions with the human Izumo1 prey. As expected, we observed that human Izumo1 did not interact with mouse Juno, but did interact with human Juno (figure 3*b*). These data suggest that the interaction between Izumo1 and Juno plays an important role in the ability of gametes from different species to recognize and fuse with each other.

## 4. Discussion

The development of IVF, where functional sperm and eggs can be isolated and successfully fused *in vitro* is a significant achievement in modern medicine which now permits infertile couples to conceive. The discovery that zona-free hamster eggs could fuse with acrosome-reacted human sperm provided an experimentally amenable model system which played an important role in the development of IVF and the functional screening of human sperm. Our recent discovery of Juno as an egg receptor for the sperm Izumo1 ligand and the demonstration that the interaction between these molecules was essential for fertilization suggested that they might provide a molecular explanation for the remarkable ability of hamster eggs to fuse with sperm from other species. Here, we have shown that the binding logic between Izumo1 and Juno proteins does indeed match the known abilities of isolated zona-free oocytes and sperm to fuse with each other *in vitro* from different mammalian species. Our findings are in agreement with the demonstration that antibodies raised against human Izumo1 prevent the ability of human sperm to fuse with hamster oocytes [10].

The prognostic ability of the hamster egg penetration test for human sperm function has been questioned [8], most probably because successful fertilization is a complex process within which gamete recognition and fusion are just individual components. By identifying the molecules which are essential for the recognition of eggs by sperm, however, it may now be possible to develop better and more cost effective diagnostic assays that do not require the use of animals to assess the quality of gametes prior to their use in IVF.
